# Comparison of piezocision and discision methods in orthodontic treatment

**DOI:** 10.1186/s40510-018-0244-y

**Published:** 2018-10-29

**Authors:** Mustafa Cihan Yavuz, Oguzhan Sunar, Suleyman Kutalmış Buyuk, Alpdogan Kantarcı

**Affiliations:** 1 Department of Periodontology, Faculty of Dentistry, Istanbul Medeniyet University, Istanbul, Turkey; 2 Department of Periodontology, Faculty of Dentistry, Ordu University, Ordu, Turkey; 3 Department of Orthodontics, Faculty of Dentistry, Ordu University, Ordu, Turkey; 4 Department of Applied Oral Sciences, Forsyth Institute, Boston, Mass USA

**Keywords:** Accelerated orthodontics, Piezocision, Discision, Orthodontic treatment

## Abstract

**Background:**

Discision method may provide an alternative to the piezocision approach in accelerated orthodontic treatment. The purpose of this study was to investigate the efficacy of discision on accelerated orthodontic tooth movement in comparison to the piezocision method in moderate crowding Angle Class I malocclusions.

**Methods:**

Thirty-five female individuals were included in this clinical study. The participants were classified into three groups as conventional fixed non-extraction orthodontic treatment only (OT, *n* = 14), piezocision in addition to fixed non-extraction orthodontic treatment (PG, *n* = 9), and discision in addition to fixed non-extraction orthodontic treatment (DG, *n* = 12). The piezocisions and discisions were performed 1 week after placement of bonding brackets. The patients were seen at 2–3 week-intervals. Initial Little’s irregularity index scores were recorded from dental casts. Periodontal parameters were measured initially, after the 1-month orthodontic treatment. Probing pocket depth, bleeding on probing, plaque index, and gingival index were recorded. Visual analog scale (VAS) was performed over the first month at different times following the bracket bonding for pain assessment. The total orthodontic treatment duration was noted.

**Results:**

The duration of orthodontic treatment was statistically decreased in PG and DG compared to OT (*P* = 0.003). There was no statistical difference between PG and DG in orthodontic treatment duration (*P* > 0.05). There was no statistical difference between the two experimental groups in terms of VAS and periodontal parameter values (*P* > 0.05).

**Conclusions:**

This is the first clinical orthodontic study to assess the effect of discision on the rate of orthodontic tooth movement. Discision is comparable to piezocision in terms of tooth movement acceleration, pain level, and periodontal status. The discision seems to be effective in reducing the time of orthodontic treatment.

## Background

The duration of orthodontic treatment may vary according to the severity of the case [1]. Decreasing the average 24-month treatment time has become an important area for clinicians and researchers [2, 3]. During the last decade, several strategies for accelerating the orthodontic treatment have been proposed. These included chemical agents, physical stimulants, and surgical procedures [4–6]. Surgical selective decortication of the alveolar bone to shorten the duration of orthodontic treatment has been used since the 1950s [7]. Initially, corticotomy was performed by open surgery with full-thickness flaps to create cortical perforations in both buccal and palatal regions as a bony block [7, 8]. The “bony block” approach led to the concept of a healing process named as regional acceleratory phenomenon (RAP) due to a reduction in bone density and increased bone turnover after surgical wounding of the bone [9]. RAP is a transient condition and does not cause permanent damage to the bones [10]. However, the original bony block and later-developed selective alveolar decortication approaches are invasive strategies posing an increased risk for root resorption and dental problems. Therefore, there is an increased desire to implement less invasive methods such as micro-osteoperforation and piezocision to achieve rapid orthodontic tooth movement [11–13].

Piezocision approach has been the most studied, minimally invasive surgical technique in accelerated orthodontic treatment [14]. Recently, the computer-guided piezocision technique was introduced as a non-invasive and safe technique to accelerate the orthodontic movement [15, 16]. However, the microvibration sound of the piezo tips may cause discomfort in some patients. As there is a certain thickness of the piezosurgery knife, there are also limited indications for use around very close-proximity roots. In addition, piezocision surgery involves the use of a device designed to perform operations on bones and is successfully used in surgical treatments; however, the availability of this device in clinics where only orthodontic patients are treated may be not available for orthodontists making it impractical in daily orthodontic treatments [12, 15].

The discision method may provide an alternative to the piezocision approach. The technique has been recently used successfully in an adolescent patient who had moderate crowding in both arches [17] and involves the use of a disc saw bur attached to a micromotor device, which is commonly used for arranging or cutting the ridge crest in dental implant surgery. Disc saws can be more ergonomic and economical than piezosurgery devices. Therefore, the purpose of this clinical study was to investigate the efficacy of discision method on accelerated orthodontic tooth movement in comparison to the piezocision method. We tested the hypothesis that there will be similar effects of these two methods on orthodontic treatment duration due to similar osteogenic impact on moderate crowding orthodontic cases.

## Methods

This study was planned as a single-center clinical trial. The study procedures were approved by the Clinic Research Ethics Local Commission of Ordu University (2018/24). The patients and their parents signed an informed consent form describing the procedures in detail. The inclusion criteria were as follows: (1) requiring fixed non-extraction orthodontic treatment, (2) full permanent dentition except third molar, (3) good oral hygiene, (4) no smoking, (5) no radiographic alveolar bone loss, (6) Class I malocclusion with moderate or severe crowding in both arches, (7) no systemic disease, and (8) no previous orthodontic and orthognathic surgery treatment.

Thirty-five female individuals were selected at the Department of Orthodontics, Faculty of Dentistry, Ordu University, Turkey. The participants were classified into three following groups: (1) patients who will receive conventional fixed orthodontic treatment (OT; *n* = 14; aged 13 to 19 years), (2) patients who will receive piezocision in addition to fixed orthodontic treatment (PG; *n* = 9; aged 13 to 18 years), and (3) patients who will receive discision in addition to fixed orthodontic treatment (DG; *n* = 12; aged 13 to 18 years). Before the orthodontic treatment, panoramic radiographs, lateral cephalometric radiographs, intra- and extra-oral photographs, and maxillary and mandibular dental casts were taken. Periodontal parameters were measured initially, after the 1-month orthodontic treatment. Probing pocket depth, bleeding on probing, plaque index, and gingival index were recorded. The study sample size was calculated by using G*Power Software version 3.1.9.2 (Universität Düsseldorf, Germany) for a reduction of the total orthodontic treatment duration with a power of 85% at the 5% significance level [13]. 

All individuals were treated with 0.022-in. slot Roth prescription self-ligated brackets. The order of orthodontic arch wires was as follows: 0.014-in., 0.016-in., 0.018-in., 0.016 × 0.022-in., 0.017 × 0.025-in. nickel-titanium arch wires were utilised for tooth alignment, and 0.019 × 0.025-in. stainless-steel arch wires were utilised for finishing stage in groups. The patients were seen at 2–3 week-intervals. The intraoral elastics were used if necessary. The orthodontic treatment was completed when adequate criteria were provided. Fixed and removable retainers were placed at the end of the orthodontic treatment. The total orthodontic treatment duration was noted. Initial Little’s irregularity index scores were performed with a digital calliper (Mitutoyo, Tokyo, Japan) on dental stone models. In PG and DG groups, piezocision and discision procedures were performed on both dental arches 1 week after placement of bonding brackets.

Visual analog scale (VAS) was performed over the first month at different times following the bracket bonding. Lateral cephalometric skeletal and dental measurements were measured on digital radiographs. Root resorptions were identified and classified on finishing radiographs.

### Piezocision procedure

Following local anaesthesia, vertical micro-incisions were performed to correspond to the centre of each interdental papilla and starting from 1 mm below the free gingival groove and passing the mucogingival line. All piezocision procedures were performed starting from teeth number 6 in both sides of the mandibular and maxillary arch. Vertical corticotomies were performed with a piezoelectric knife (Mectron Piezosurgery Device, Mectron, Genova, Italy) approximately 7 mm in length and 3 mm in depth. There was no need for suturing the incision lines; all piezocision procedures were done flapless. Irrigation was used in piezocision procedure. No hard or soft tissue grafts were applied to the vestibular region of the teeth. The patients were advised to take analgesics such as paracetamol if necessary.

### Discision procedure

**Fig. 1:**
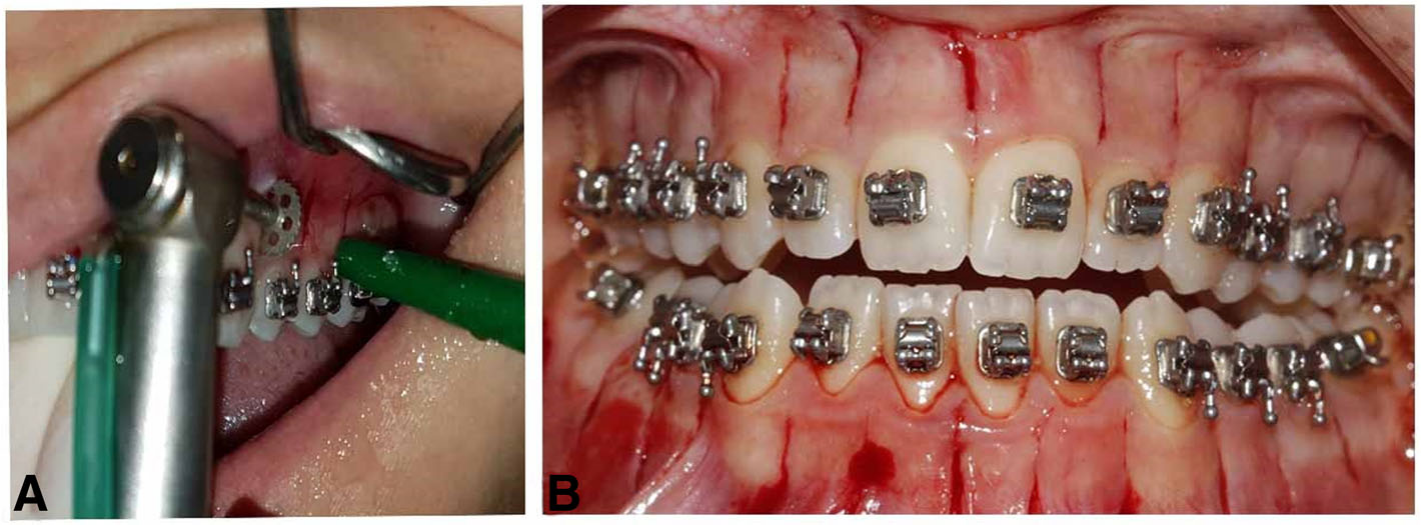
**a** Intraoral discision application. **b** Discision post-operative view

The disc incision protocol was performed as defined [17]. Following local anaesthesia, vertical micro-incisions were performed to correspond to the centre of each interdental papilla and starting at 1 mm below the free gingival groove and crossing the mucogingival line. Vertical corticotomies were then performed with a disc saw (Osstem Implant, Esset KIT-Saw, Seoul, Korea) approximately 7 mm in length and 3 mm in depth. There was no need for flap elevation or suturing (Fig. 1). Also, irrigation was used in discision procedure. The patients were advised to take analgesics such as paracetamol if necessary.

### Statistical analyses

All data parameters were statistically analysed by using the SPSS (SPSS for Windows version 20.0; SPSS Inc., Chicago, IL) program. After performing the normal distribution test, parametric tests were performed to the parameters having a normal distribution, while non-parametric tests were performed to the parameters with non-normal distributions. The Shapiro-Wilk test was performed to test the data for normal distribution. The data were analysed by using one-way analysis of variance, Kruskal-Wallis, Mann-Whitney *U* test, and independent *t* tests. Post hoc LSD test was used for parametric variables, and the Mann-Whitney *U* test was used for non-parametric variables.

## Results

This study consisted of three groups. There was a statistical homogeneity in terms of age distribution in all three groups (*P* > 0.05). The initial Little’s irregularity index scores of the study groups are shown in Table 1. All groups were statistically homogeneous in terms of dental crowding scores in both arches.

**Table 1:** Initial Little’s irregularity index score of the groups

Groups	Maxillary Little’s score	Mandibular Little’s score
Discision + orthodontic treatment	6.86 (3.76)	6.14 (3.79)
Piezocision + orthodontic treatment	10.48 (6.16)	6.47 (3.87)
Orthodontic treatment only	8.65 (3.48)	6.72 (2.53)
*P*	.247*	.906**

*Results of Kruskal-Wallis test; **results of one-way ANOVA test

Periodontal measurements of study groups are shown in Tables 2 and 3. There was no statistical difference between groups in any parameters. The VAS values in the PG and DG groups are shown in Tables 4 and 5. There was no statistical difference between the two experimental groups in terms of VAS values (*P* > 0.05).

**Table 2:** Comparison of maxillary periodontal parameters in experimental groups according to different treatment periods

Variables	T0	*P*	T1	*P*
PPD				
Discision + orthodontic treatment	2.08 (1.96)	.688*	2.21 (0.18)	.480*
Piezocision + orthodontic treatment	2.11 (0.18)		2.28 (0.24)	
BOP				
Discision + orthodontic treatment	5.69 (5.07)	.321*	3.18 (3.82)	.148**
Piezocision + orthodontic treatment	8.39 (7.11)		6.70 (5.72)	
Plaque index				
Discision + orthodontic treatment	0.62 (0.32)	.536*	0.44 (0.22)	.434*
Piezocision + orthodontic treatment	0.71 (0.34)		0.54 (0.34)	
Gingival index				
Discision + orthodontic treatment	0.41 (0.56)	.164**	0.42 (0.80)	.474**
Piezocision + orthodontic treatment	0.56 (0.38)		0.48 (0.48)	

*PPD* probing pocket depth, *BOP* bleeding on probing, *T0* before orthodontic treatment, *T1* 1 month after discision/piezocision procedure. *Results of independent *t* test, **results of Mann-Whitney *U* test

**Table 3:** Comparison of mandibular periodontal parameters in experimental groups according to different treatment periods

Variables	T0	*P*	T1	*P*
PPD				
Discision + orthodontic treatment	1.93 (0.08)	.294*	2.03 (0.20)	.355**
Piezocision + orthodontic treatment	2.00 (0.18)		2.13 (0.19)	
BOP				
Discision + orthodontic treatment	6.84 (5.74)	.972**	5.28 (4.69)	.930*
Piezocision + orthodontic treatment	6.82 (5.69)		5.09 (5.09)	
Plaque index				
Discision + orthodontic treatment	0.73 (0.39)	.487*	0.56 (0.26)	.886*
Piezocision + orthodontic treatment	0.61 (0.33)		0.54 (0.32)	
Gingival index				
Discision + orthodontic treatment	0.45 (0.41)	.255**	0.36 (0.23)	.859**
Piezocision + orthodontic treatment	0.53 (0.33)		0.46 (0.42)	

*PPD* probing pocket depth, *BOP* bleeding on probing, *T0* before orthodontic treatment, *T1* 1 month after discision/piezocision procedure. *Results of independent *t* test, **results of Mann-Whitney *U* test

**Table 4:** Comparison of maxillary VAS scores within experimental groups after the accelerated procedures according to different observation periods

Variables	VAS score	*P*
4 h		
Discision + orthodontic treatment	2.00 (1.00–7.00)	.701*
Piezocision + orthodontic treatment	3.00 (1.00–5.00)	
24 h		
Discision + orthodontic treatment	0.00 (0.00–1.00)	.897*
Piezocision + orthodontic treatment	0.00 (0.00–2.00)	
3 days		
Discision + orthodontic treatment	0.00 (0.00–0.00)	.744*
Piezocision + orthodontic treatment	0.00 (0.00–1.00)	
7 days		
Discision + orthodontic treatment	0.00 (0.00–0.00)	.109*
Piezocision + orthodontic treatment	0.00 (0.00–1.00)	
30 days		
Discision + orthodontic treatment	0.00 (0.00–0.00)	.269*
Piezocision + orthodontic treatment	0.00 (0.00–0.00)	

*Mann-Whitney *U* test

**Table 5:** Comparison of mandibular VAS scores within experimental groups after the accelerated procedures according to different observation periods

Variables	VAS score	*P*
4 h		
Discision + orthodontic treatment	2.00 (2.00–4.00)	.511*
Piezocision + orthodontic treatment	3.00 (1.50–6.00)	
24 h		
Discision + orthodontic treatment	1.00 (0.00–2.00)	.512*
Piezocision + orthodontic treatment	0.00 (0.00–1.00)	
3 days		
Discision + orthodontic treatment	0.00 (0.00–0.00)	.827*
Piezocision + orthodontic treatment	0.00 (0.00–0.00)	
7 days		
Discision + orthodontic treatment	0.00 (0.00–0.00)	.827*
Piezocision + orthodontic treatment	0.00 (0.00–0.00)	
30 days		
Discision + orthodontic treatment	0.00 (0.00–0.00)	1.000*
Piezocision + orthodontic treatment	0.00 (0.00–0.00)	

*Mann-Whitney *U* test

The orthodontic treatment durations of all groups are shown in Table 6. The duration of orthodontic treatment was statistically decreased in PG and DG compared to OT (*P* = 0.003). There was no statistical difference between PG and DG in orthodontic treatment duration (*P* > 0.05).

**Table 6:** Comparison of orthodontic treatment duration among the groups

Groups	Orthodontic treatment duration-day	P*	Post hoc rests		
			DG-PG	DG-OT	PG-OT
Discision + orthodontic treatment	209.580 (73.50)	.003	.255	.002	.011
Piezocision + orthodontic treatment	238.56 (69.90)				
Orthodontic treatment only	324.50 (81.65)				

^*^Results of Kruskal-Wallis test

## Discussion

In this study, we compared the efficacy of the discision method to the piezocision in accelerating the orthodontic tooth movement. Both methods significantly enhanced the rate of orthodontic treatment compared to the conventional approach with no significant difference between them suggesting that discision approach could be a cheaper alternative to the piezocision in rapid orthodontics.

Various factors can affect the quality and rate of orthodontic tooth movement [18]. Age [19], sex hormones [20], and occlusal forces [21] can alter the speed of tooth movement by affecting bone density and remodelling. Alikhani et al. [18] stated the gender distribution as an important factor while Charavet et al. [13] identified the age as critical for the outcomes. Patients with moderate or severe crowding associated with Class I or II are the most appropriate cases for corticotomy indications [14]. To eliminate these potential confounding variables, we distributed our patients with similar age range only female subjects and selected our patients among those with Angle Class I malocclusion in non-extraction orthodontic treatment groups.

Patients included in the study were periodontally healthy and there was no statistical difference in periodontal status between groups. This is an expected outcome for patients with cooperation and good oral hygiene and is consistent with the results of other studies [13]. Orthodontic tooth movement is one of the causes of gingival recession. It is not known whether rapid tooth movement increases gingival recession. Charavet et al. [13] reported that overall recession scores did not increase after treatment in both piezocision group and control group. The results of gingival recession scores in our study were consistent with this study. The gingival recession that existed prior to treatment in 3 of the 24 patients, in 2 from the control group, and in 1 from the piezocision group increased during orthodontic treatment. This increase in initial gingival recessions may be related to the bone topography and whether the positioning of the teeth regardless of whether the orthodontic treatment is conventional or there is a rapid tooth movement. Casetta et al. [22] treated ten patients with severe dental crowding with clear aligners and corticotomy-facilitated orthodontics, and they found that there was no difference between the pre-treatment and post-treatment periodontal indices. In our study, there was no statistical difference between experimental groups in any periodontal parameters.

Piezocision is widely used as a selective decortication method in association with successful and rapid tooth movement. Yet, according to a recently published systematic review, there is only one study related to piezocision applied to the entire maxillary and mandibular dental arch [23]. Charavet et al. [13] reported that the treatment of the piezocision group was 43% faster than that of the control group comparable to our 27% reduction in treatment time with piezocision. The above-mentioned study had the rate of crowding less than that of our study; therefore, it is possible that the piezocision in our study had a higher rate of tooth movement. The shorter treatment time may also be due to the younger age range of the participants included in the study.

**Fig. 2:**
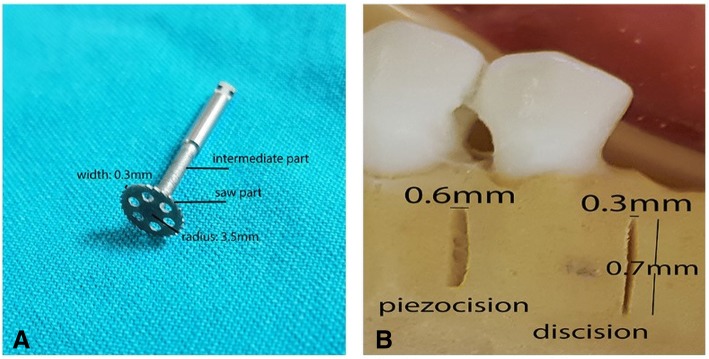
**a** Disc saw. **b** Comparison of piezocision and discision in a dental study model

**Fig. 3:**
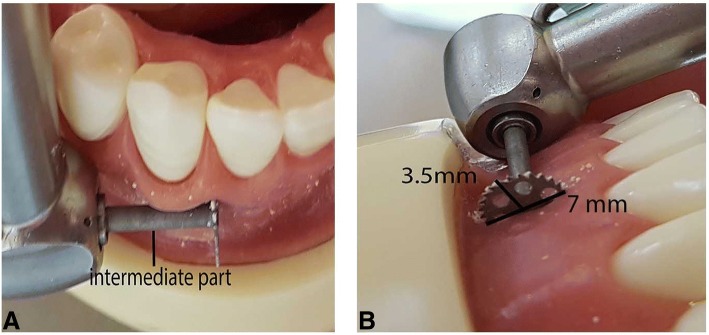
**a** Controlled entry with a maximum depth of 3 mm. **b** Single entry-point with a 7 mm length

The discision method was recently introduced as a case report [17]. The authors suggested that the discision method shortened the duration of treatment by performing rapid tooth movement. The present study is the first clinical study evaluating the effect of the discision method on rapid orthodontic tooth movement. Thus, we could not identify an article in the literature to compare the DG results of our study. Our data demonstrated that the discision method accelerates orthodontic tooth movement by 35.5%. The technique is performed with a disc-shaped saw-bur, which is placed on a micromotor. This saw-bur is normally used for bone augmentation in implant dentistry. The blade thickness of the disc saw and piezosurgery knife are 0.3 mm and 0.6 mm, respectively (Fig. 2). As the disc saw is two times thinner than the piezosurgery knife, it may provide more reliable indications for flapless corticotomies, which are already a risky procedure between close adjacent roots, especially for crowded mandibular incisors. In piezocision studies, it is suggested that an incision line should be formed with an average length of 5–8 mm at a depth of 3 mm. Since the disc saw that we use has a 3.5-mm radius and the main body will act as a stopper, we can form the desired 3-mm-deep incision line in a more controlled and practical way than in the piezocision method. In addition, since the disc saw has a diameter of 7 mm, an incision line of the desired length can be formed at a single entry-point (Fig. 3). The discision method was twice as much faster than the piezocision method. Thus, application of the discision method in a shorter time can be considered an advantage of this method.

The evaluation of root resorption after orthodontic tooth movement is important. No study evaluating root resorption after acceleration techniques reported significant root shortening compared to the conventional approaches [13, 21, 23]. In fact, Shoreibah et al. [24] reported less resorption of the root in the corticotomy group than in the control group. This result is not surprising, as the root will encounter relatively less resistance in the process of rapid tooth movement that is known to occur due to the temporary diminution of bone density. In our study, both methods of acceleration resulted in similar root resorption rates compared to the conventional tooth movement.

A limitation of this study was that cone-beam computed tomography was not used to examine discision and piezocision cuts. Although cone-beam computed tomography is a reliable method in three-dimensional imaging, we did not prefer it to not give the patients extra radiation doses. Another limitation of this study was that disc saw may damage alveolar soft tissues, so the operator must consider it.

## Conclusions

This was the first clinical trial to assess the effect of discision method on the rate of orthodontic tooth movement. In this study, irregularity index, periodontal status, pain, and duration of orthodontic treatment were focused and compared between groups. We have shown that this technique successfully facilitated rapid tooth movement. The discision method can be used in daily orthodontic practice because the disc saw is much cheaper than the piezosurgery device, it is easy to carry, and most importantly, the disc saw is twice as thin as the piezosurgery knife. The efficacy of discision procedure must be confirmed in more numerous controlled clinical trials.

## Abbreviations

DG: Discision in addition to fixed orthodontic treatment
OT: Conventional fixed orthodontic treatment
PG: Piezocision in addition to fixed orthodontic treatment
VAS: Visual analog scale
